# Association between systemic blood pressure control and outcomes of Conbercept treatment for macular edema secondary to retinal vein occlusion

**DOI:** 10.3389/fmed.2026.1769047

**Published:** 2026-04-10

**Authors:** Qin Wu, Bin Gong, Hui Zhang, Yuan Tao, Chenming Zhang, Guanghui Li, Nana Yang

**Affiliations:** Department of Ophthalmology, Jinan Second People’s Hospital, Jinan, Shandong, China

**Keywords:** blood pressure, Conbercept, macular edema, retinal vein occlusion, visual acuity

## Abstract

**Objective:**

To evaluate the association between the level of systemic blood pressure (BP) control and the clinical outcomes of intravitreal Conbercept for macular edema (ME) secondary to retinal vein occlusion (RVO).

**Methods:**

We conducted a retrospective study of hypertensive patients who were treated between January and June 2025 at the Second People’s Hospital of Jinan for ME secondary to RVO. All patients received a 0.05 mL (10 mg/mL) intravitreal Conbercept injection and concurrent medical management for hypertension. Patients were stratified into three groups depending on if their BP were under control (Group A), partially under control (Group M), or out of control (Group Z) based on their BP during the follow-up over 1 month.

**Results:**

The study included 76 patients (36 males, 40 females) with a mean age of 63.11 ± 10.10 years. The number of patients in each group was as follows: Group A, *n* = 25; Group M, *n* = 31; Group Z, *n* = 20. A total of 76 eyes were analyzed, of which 34 had central RVO and 42 had branch RVO. At 1 month post-treatment, Best Corrected Visual Acuity (BCVA) was significantly better in Groups A and M compared to Group Z (*p* < 0.001). Foveal thickness was significantly lower in Groups A and M compared to Group Z at 1 week and 1 month (both *p* < 0.001). No significant differences in BCVA or foveal thickness were found between Group A and Group M at any time point.

**Conclusion:**

Systemic BP control is associated with the short-term efficacy of Conbercept for ME secondary to RVO, and patients with well-controlled BP achieve superior visual and anatomical outcomes. This underscores the importance of co-managing systemic hypertension.

## Introduction

Retinal vein occlusion (RVO) is a common retinal vascular disorder and the second leading cause of vision loss from vascular disease, surpassed only by diabetic retinopathy (1). It represents a significant public health burden, frequently resulting in severe and permanent vision impairment, which substantially impacts patient quality of life (2). The most common and vision-threatening complication of RVO is macular edema (ME), which represents the primary cause of central vision loss in affected patients (3). This high prevalence of ME necessitates a clear understanding of both the intraretinal mechanisms that drive the edema and the systemic factors that may influence its progression and treatment response.

The pathophysiology of ME secondary to RVO is primarily driven by venous occlusion, which leads to retinal ischemia and hypoxia. This ischemic state triggers the upregulation of inflammatory mediators and vascular endothelial growth factor (VEGF) (4, 5). VEGF, in turn, increases the phosphorylation of tight junction proteins, which disrupts the integrity of the blood-retina barrier. This disruption causes vascular hyper-permeability, leading to the extravasation of fluid and plasma components into the macula (6, 7). This pathophysiological understanding has established intravitreal anti-VEGF agents as the first-line therapy for RVO-related ME (8, 9). The major classes of anti-VEGF drugs currently used for retinal (fundus) conditions include monoclonal antibodies (e.g., Ranibizumab), fusion proteins (e.g., Aflibercept and Conbercept), and bispecific antibodies (e.g., Fareximab) (10). Conbercept, a recombinant fusion protein that binds all VEGF-A isoforms and placental growth factor, has demonstrated efficacy in reducing foveal thickness and improving visual acuity in these patients (11, 12). It was approved by the China Food and Drug Administration in 2013 and entered the Chinese market in 2014. As of March 2026, it has not received FDA or EMA approval and remains primarily commercialized in China.

However, RVO is rarely an isolated ocular event. It is strongly associated with a spectrum of systemic vascular risk factors, including hypertension, diabetes, hyperlipidemia, and chronic kidney disease (13, 14). Among these, systemic hypertension is one of the most prevalent and critical contributing factors (15). Chronic hypertension independently damages the vascular endothelium and alters microvascular permeability (7, 16). This creates a “dual-assault” on the retinal vasculature: The acute ischemic event of RVO is superimposed on a microvascular system already compromised by chronic hypertension. While anti-VEGF therapy targets the *consequences* of RVO (i.e., VEGF upregulation), the influence of managing the *underlying* systemic risk factors on treatment efficacy is less clear. Some case reports have even documented the resolution of RVO-associated ME with strict hypertension control *alone*, underscoring the profound impact of systemic health on this ocular condition (17). Therefore, this study aims to investigate the effect of systemic blood pressure (BP) control on the visual and anatomical outcomes of intravitreal Conbercept treatment for macular edema secondary to retinal vein occlusion.

## Methods

### Patients

We retrospectively reviewed patients who (1) visited the Second People’s Hospital of Jinan between January and June 2025, (2) had hypertension complicated by RVO secondary to macular edema, and (3) received intravitreal injection of Conbercept. All included patients had hypertension (systolic BP ≥ 140 mmHg or diastolic BP ≥ 90 mmHg), had a foveal thickness of >400 μm (as determined by optical coherence tomography, OCT), met the diagnostic criteria for central or branch retinal vein occlusion (as determined by wide-field fundus photography and fundus fluorescein angiography), and had never previously received an intravitreal injection in the affected eye. Patients were excluded if they had normal BP (<140/<90 mmHg) at the time of consultation, were diabetic, or had other eye diseases (e.g., corneal disease, cataracts, glaucoma, vitreous hemorrhage, macular hole, other refractive media opacities, as determined by slit lamp examination).

This study was approved by the Research Ethics Committee of the Second People’s Hospital of Jinan (approval No. JNEYE20250628) and complied with the principles of Declaration of Helsinki.

### Examinations

Baseline indicators were measured as follows. Patients took oral antihypertensive medication as prescribed, came to the hospital between 8 and 9 a.m., and rested for 30 min before the measurements. The patient sat upright, and the BP in the upper right arm was measured with a calibrated arm-type electronic BP monitor. The average value of three measurements was taken. The measurement of BP preceded all eye exams.

Best corrected visual acuity (BCVA) was tested using the international standard visual acuity chart. The affected eye was examined using a slit-lamp microscope with standard magnifications and illumination settings. Intraocular pressure was measured by Goldmann applanation tonometry according to standard clinical technique. The macula and optic nerve were imaged using a StratusOCT device (Zeiss, Germany). To reduce measurement bias, BCVA, slit-lamp, and OCT examinations were all performed by a single physician who was not involved in the surgical treatment or group allocation. The dilated fundus was examined using a binocular indirect ophthalmoscope and a condensing lens to obtain a wide-field stereoscopic view of the posterior segment. After the patient received intravenous fluorescein, sequential fundus photographs were obtained to document retinal and choroidal vascular filling and leakage. This fundus fluorescein angiography (FA) was performed by a specialist technician who was also not involved in the study grouping. While baseline FA was performed to characterize the vascular leakage patterns and confirm the diagnosis of ME, diagnosis and follow-up were primarily guided by OCT imaging, especially in cases with significant intraretinal hemorrhage where FA was limited by masking effects.

### Treatment and follow-up

Before the surgery, an internal medicine consultation was requested to assist in controlling BP, and written informed consent from the patient was collected. Three days prior to treatment, the patient was administered levofloxacin eye drops (5 mL:24.4 mg, Yangtze River Pharmaceutical Group, NMPA H20203092) to the affected eye four times daily. This 3-day course of preoperative topical levofloxacin was required by the institutional safety protocol. Before the treatment, the operated eye was fully dilated with compound tropicamide eye drops (5 mL:25 mg/25 mg tropicamide/phenylephrine hydrochloride, Changchun Dirui Pharmaceutical Co., Ltd., NMPA H20103127), and surface anesthesia was performed with proparacaine hydrochloride eye drops (0.4 mL/2 mg, Unither Pharmaceuticals, NMPA H20103352). The surgical area was routinely disinfected, sterile surgical drapes were laid, eyelids were opened with an eyelid speculum, and the conjunctival sac was irrigated with povidone-iodine. The medication was injected using a 27G needle and a disposable sterile syringe specific for intravitreal injections. The needle was vertically inserted into the vitreous body 3.5 mm posterior to the corneal limbus in the inferior temporal region (18), and 0.05 mL (10 mg/mL) Conbercept ophthalmic injection (0.20 mL, Chengdu Kanghong Pharmaceutical Group Co., Ltd., NMPA SJ20170003) was injected. The needle was withdrawn, and the injection site was compressed with a sterile cotton swab for 1 min. Tobramycin and dexamethasone eye ointment (3.5 g:10.5 mg/3.5 mg tobramycin/dexamethasone, Alcon; NMDA HJ20181126) was applied to the conjunctival sac, and the operated eye was covered with a sterile eye pad.

Following the treatment, levofloxacin eye drops were administered for 1 week. BCVA and BP were measured at 1 day, 1 week, and 1 month post-treatment according to the same protocol. The first follow-up at 1 day post-injection was in strict accordance with the *Quality Control Standards for Intravitreal Injection in China* (19) and the *Expert Consensus on the Establishment and Management of One-stop Intravitreal Injection Mode* (18). The primary clinical rationale for this immediate review was to conduct a safety screening via slit-lamp examination to rule out acute post-operative complications such as endophthalmitis or severe intraocular inflammation. At this visit, BCVA and OCT were performed to document early anatomical changes in foveal thickness and establish a post-treatment baseline. During subsequent visits at 1 week and 1 month, BCVA and OCT were repeated to dynamically monitor ocular recovery and macular morphology.

### Statistical analysis

All analyses were performed in R 4.5.3. Continuous variables were summarized as mean ± SD or median [P25, P75] based on the Shapiro–Wilk normality test. Categorical variables were reported as counts (percentages). For cross-sectional comparisons, we employed one-way ANOVA (with Welch’s correction for unequal variances) or the Kruskal–Wallis test, followed by Bonferroni-adjusted post-hoc comparisons. Categorical data were analyzed using Pearson’s *χ*^2^ or Fisher’s exact tests. To evaluate the primary outcomes across time points, longitudinal comparisons initially adopted repeated-measures ANOVA (RM-ANOVA) to test Time, Group, and Group × Time interaction effects. Sphericity was assessed using Mauchly’s test, and Greenhouse–Geisser corrections were applied where the assumption of sphericity was violated. Bonferroni-adjusted within-group contrasts were analyzed to compare follow-up results against baseline.

To further account for the repeated-measures structure and enable multivariable adjustment, we employed linear mixed models (LMM) using the *lmerTest* package. The models included fixed effects for Group, Time, and their interaction, with random intercepts per subject to account for within-patient correlation. *p*-values and denominator degrees of freedom were calculated using the Satterthwaite approximation. Multivariable LMMs were constructed to adjust for potential confounders, including age, sex, baseline intraocular pressure (IOP), RVO type (central vs. branch), RVO duration, and hypertension duration. The primary inferential analysis was LMM, and cross-sectional comparisons at individual time points were considered secondary descriptive analyses. Because baseline values of the dependent variables were already integrated into the repeated-measures structure of the models (as the “Time 0” data point), to avoid collinearity, we did not include them as separate fixed-effect covariates. Instead, to evaluate the cross-domain impact of baseline disease severity, we included baseline FT as a covariate in the BCVA model, and baseline BCVA as a covariate in the FT model. This approach allows the model to determine, for example, whether the initial degree of anatomical edema independently limits the potential for functional visual recovery, regardless of BP control.

This adjustment is essential to account for the ceiling effect in visual recovery and the statistical phenomenon of regression to the mean, whereby patients with more severe baseline edema often demonstrate numerically larger reductions in thickness regardless of other factors.

Adjusted mean differences (estimated marginal means) and 95% confidence intervals (CIs) were calculated using the *emmeans* package with Bonferroni correction. To assess the study’s sensitivity to detect clinically meaningful differences, the minimal detectable effect (MDE) was calculated for pairwise group contrasts at 80% power. Longitudinal trend visualizations were generated using the *ggplot2* package. Differences were considered statistically significant when *p* < 0.05 (two-tailed).

## Results

### Patient characteristics

A total of 76 patients (76 eyes) were included, comprising 34 (44.7%) with central retinal vein occlusion and 42 (55.3%) with branch retinal vein occlusion. The cohort included 36 males (47.4%) and 40 females (52.6%), and their age was 63.11 ± 10.10 years (range: 44–88). The history of hypertension was 11.35 ± 2.82 years (range: 5.1–17.9), and the duration of RVO was 5.71 ± 1.01 months (range: 3.4–7.8). The duration of RVO refers to the time from the patient’s self-reported onset of visual symptoms to their first presentation and treatment at our tertiary center; all included patients were treatment-naive, and this interval reflects real-world delays in referral and diagnosis. At baseline, the IOP was 16.6 [15.8, 17.2] mmHg (range: 15.0–18.3), the overall BCVA was 1.14 [0.84, 1.28] LogMAR (range: 0.68–1.45), and the foveal thickness was 500.56 ± 42.42 μm (range: 362.54–604.08).

Patients were stratified into three groups based on BP control during the one-month follow-up:

- Group A (Controlled; *n* = 25): Normal BP (<140/<90 mmHg) at all three follow-ups.
- Group M (Partially Controlled; *n* = 31): Normal BP at one or two follow-up points.
- Group Z (Uncontrolled; *n* = 20): High BP (≥140/≥90 mmHg) at all three follow-ups.

As shown in Table 1, there were no statistically significant differences among the three groups at baseline regarding age, sex, BCVA, foveal thickness, hypertension duration, RVO duration, or RVO type (all *p* > 0.05). There were no missing data or loss to follow-up.

**Table 1:** Patient information.

Parameter	Group A (*n* = 25)	Group M (*n* = 31)	Group Z (*n* = 20)	Statistic	*p*
Age (years)	63.94 ± 11.66	62.07 ± 8.79	63.67 ± 10.28	*F* = 0.275	0.760
Sex (M/F)	12/13	15/16	9/11	*χ*^2^ = 0.062	0.970
Baseline measures					
BCVA (LogMAR)^†^	1.08 [0.85, 1.28]	1.12 [0.82, 1.28]	1.20 [0.85, 1.33]	*H* = 1.315	0.518
IOP (mmHg)^†^	16.65 ± 0.90	16.53 ± 0.96	16.43 ± 0.91	*F* = 0.31	0.734
FT (μm)^†^	503.70 [477.08, 521.39]	511.37 [467.16, 531.08]	504.19 [486.09, 515.24]	*H* = 0.242	0.886
Disease history					
Hypertension (years)	11.30 ± 2.87	11.55 ± 2.53	11.10 ± 3.26	*F* = 0.161	0.852
RVO (months)	5.69 ± 1.16	5.73 ± 0.94	5.69 ± 0.96	*F* = 0.013	0.987
Type of RVO^†^				*χ*^2^ = 0.119	0.942
Central	11	12	11		
Branch	14	19	9		

^†^BCVA, best corrected visual acuity; IOP, intraocular pressure, FT, foveal thickness; RVO, Retinal Vein Occlusion.

### Visual acuity outcomes

**Figure 1:**
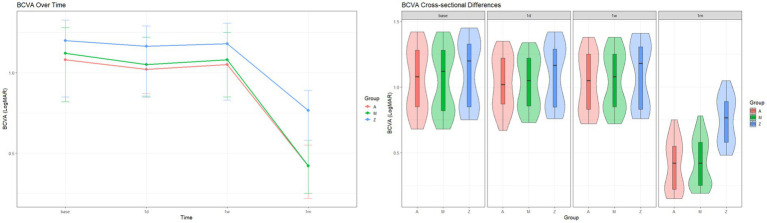
Changes of BCVA (unit: LogMAR) after treatment.

Across the three groups, there was significant difference in BCVA only at 1 month (*χ*^2^ = 27.1, *p* < 0.001). Post-hoc analysis revealed that at 1 month post-surgery, compared to Group Z, BCVA was significantly better in Group A (*W* = 50, adjusted *p* < 0.001) and Group M (*W* = 73, adjusted *p* < 0.001), but there was no significant difference between Group A and Group M (*W* = 340, adjusted *p* = 1.000) (Figure 1).

In RM-ANOVA, a significant Group-by-Time interaction was observed for (*F* = 97.3, adjusted *p* < 0.001, Table 2), indicating that the pattern of change over time differed significantly among the three groups. For Group A, BCVA showed significant improvement at 1 week (*W* = 62.5, adjusted *p* = 0.007) and 1 month (*t* = −51.6, adjusted *p* < 0.001) post-injection. For Group M, BCVA showed significant improvement only at 1 month post-injection (*W* = 0, adjusted *p* < 0.001). For Group Z, BCVA showed significant improvement at all three follow-ups (1 day, *W* = 33, adjusted *p* = 0.021; 1 week, *W* = 30.5, adjusted *p* = 0.005; 1 month, *W* = 0, adjusted *p* < 0.001).

**Table 2:** Comparison of BCVA (unit: LogMAR).

Time	Group A (*n* = 25)	Group M (*n* = 31)	Group Z (*n* = 20)	Statistic	*p*
Baseline	1.08 [0.85, 1.28]	1.12 [0.82, 1.28]	1.20 [0.85, 1.33]	*H* = 1.32	0.518
After surgery					
1 day	1.02 [0.87, 1.22]	1.05 [0.85, 1.22]	1.16 [0.85, 1.29]^#^	*H* = 1.47	0.479
1 week	1.05 [0.83, 1.25]^##^	1.08 [0.85, 1.25]	1.18 [0.83, 1.31]^##^	*H* = 1.67	0.433
1 month	0.42 [0.22, 0.55]^###^***	0.42 [0.25, 0.58]^###^***	0.76 [0.58, 0.89]^###^	*H* = 27.1	<0.001
Group				*F* = 2.64	0.078
Time				*F* = 3,530	<0.001
Group × Time				*F* = 97.3	<0.001

^#^Adjusted *p* < 0.05, ^##^adjusted *p* < 0.01, ^###^adjusted *p* < 0.001, compared to baseline within the same group (Bonferroni post-hoc test). ^***^Adjusted *p* < 0.001 compared to Group Z at the same time point (Bonferroni post-hoc test).

### Anatomical outcomes

**Figure 2:**
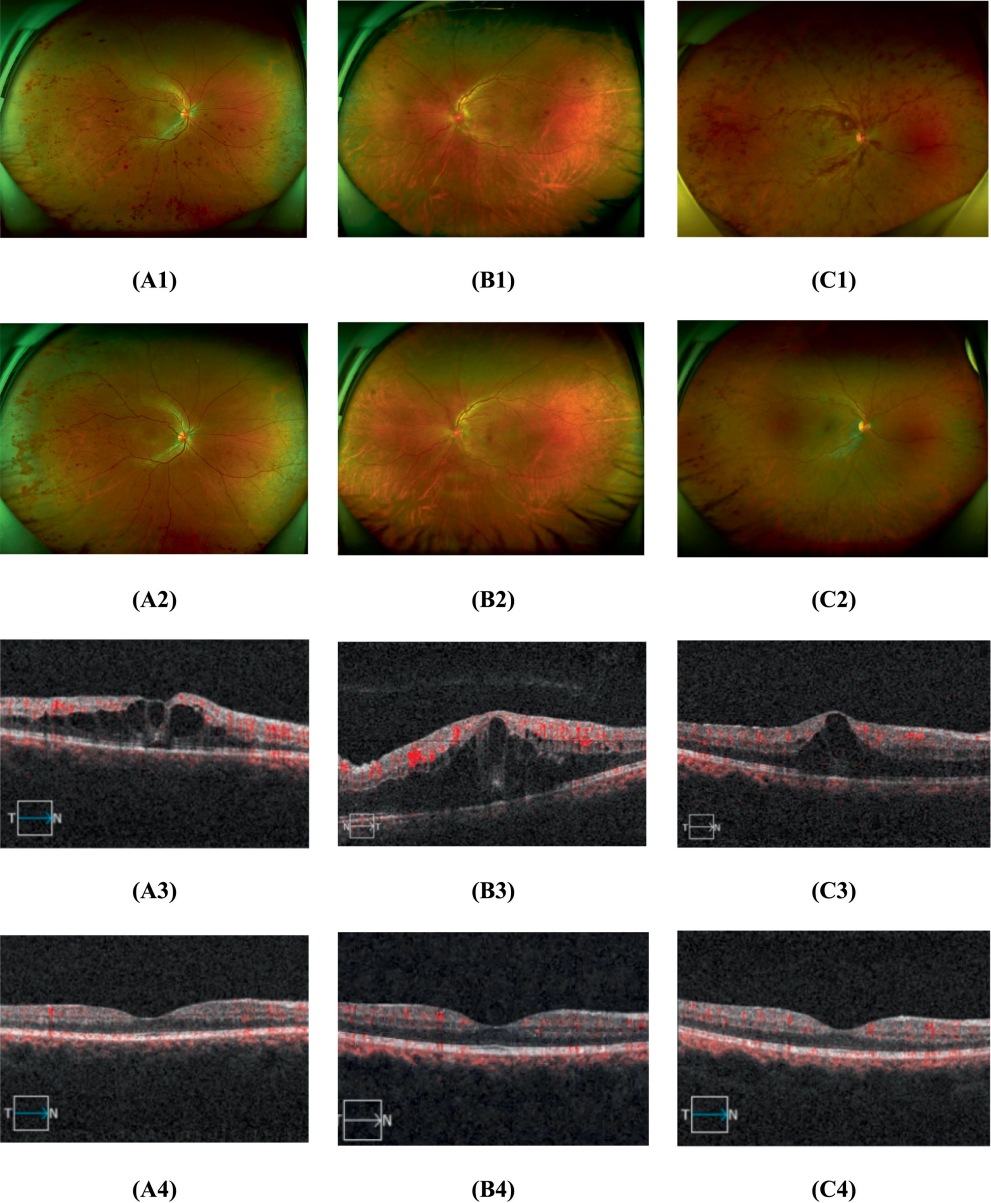
Representative fundus photographs and macular OCT images at baseline (1, 3) and after 1 month (2, 4). **(A)** Group A, **(B)** Group M, **(C)** Group Z.

**Figure 3:**
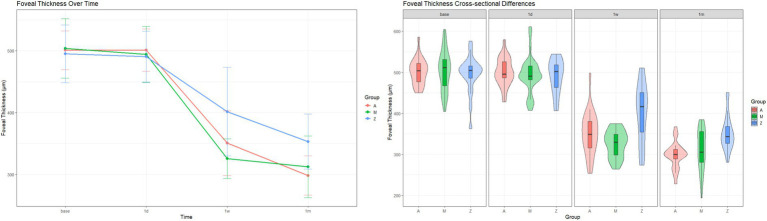
Changes of foveal thickness (unit: μm) after treatment.

Figure 2 shows representative fundus photographs and macular OCT images at baseline and after 1 month. Across the three groups, significant differences in foveal thickness were observed at 1 week (*F* = 10.7, *p* < 0.001) and 1 month (*F* = 10.7, *p* < 0.001). At 1 week, foveal thickness was significantly lower in Group A (*t* = −2.67, adjusted *p* = 0.035) and Group M (*t* = −4.47, adjusted *p* < 0.001) than in Group Z, but the difference between Group A and Group M was not statistically significant (*t* = 2.08, adjusted *p* = 0.133). This pattern persisted at 1 month: Both Group A (*t* = −4.66, adjusted *p* < 0.001) and Group M (*t* = −3.06, adjusted *p* = 0.011) had significantly lower foveal thickness than Group Z, and again, the difference between Group A and Group M was not significant (*t* = −1.28, adjusted *p* = 0.616) (Figure 3).

Regarding longitudinal development, a significant Group-by-Time interaction was observed in RM-ANOVA (*F* = 6.30, adjusted *p* < 0.001). For all groups, significant improvement was noted only at 1 week and 1 month post injection (Group A, 1 week, *t* = −12.1, adjusted *p* < 0.001, 1 month, *t* = −23.1, adjusted *p* < 0.001; Group M, 1 week, *t* = −16.2, adjusted *p* < 0.001, 1 month, *t* = −16.4, adjusted *p* < 0.001; Group Z, 1 week, *t* = −5.42, adjusted *p* < 0.001, 1 month, *W* = 3, adjusted *p* < 0.001).

### Multivariable adjusted analysis

Multivariable LMM was employed as the primary analysis to confirm the associations identified in the descriptive group comparisons at individual time points (Tables 2, 3). The LMM adopted restricted maximum likelihood (REML) estimation and controlled for baseline characteristics and cross-domain clinical confounders (Table 4). Specifically, the BCVA model adjusted for baseline FT, while the FT model adjusted for baseline BCVA, ensuring that the independent effect of BP control was evaluated relative to the initial severity of both anatomical and functional damage. The LMM explained a high proportion of variance in both outcomes (BCVA: conditional *R*^2^ = 0.987, marginal *R*^2^ = 0.603; FT: conditional *R*^2^ = 0.791, marginal *R*^2^ = 0.779). After adjusting for age, sex, RVO type, and disease duration, the Group × Time interaction remained highly significant for BCVA and foveal thickness (both *p* < 0.001). Notably, RVO type was not a significant predictor of either BCVA (*p* = 0.744) or foveal thickness (*p* = 0.754).

**Table 3:** Comparison of macular foveal thickness (unit: μm).

Time	Group A (*n* = 25)	Group M (*n* = 31)	Group Z (*n* = 20)	Statistic	*p*
Baseline	503.70 [477.08, 521.39]	511.37 [467.16, 531.08]	504.19 [486.09, 515.24]	*H* = 0.242	0.886
After surgery					
1 day	495.87 [486.42, 526.07]	490.55 [481.78, 515.28]	501.98 [462.86, 518.56]	*H* = 0.776	0.678
1 week	350.39 ± 52.39^###,*^	325.55 ± 31.93^###,***^	401.43 ± 71.53^###^	*F* = 10.7	<0.001
1 month	298.22 ± 31.64^###,***^	312.26 ± 49.72^###,*^	353.07 ± 44.42^###^	*F* = 10.7	<0.001
Group				*F* = 7.84	<0.001
Time				*F* = 327	<0.001
Group × Time				*F* = 6.30	<0.001

^###^Adjusted *p* < 0.001 compared to baseline within the same group (Bonferroni post-hoc test). ^*^Adjusted *p* < 0.05, ^***^adjusted *p* < 0.001, compared to Group Z at the same time point (Bonferroni post-hoc test).

**Table 4:** Multivariable linear mixed model for visual and anatomical outcomes.

Variable^†^	BCVA		FT	
	*F* ^§^	*P*	*F* ^§^	*P*
Fixed effects				
Group	2.11	0.129	8.10	<0.001
Time	3529.77	<0.001	327.30	<0.001
Group × Time	97.32	<0.001	6.30	<0.001
Covariates				
Baseline FT	1.78	0.187	–	–
Baseline BCVA	–	–	0.54	0.464
Age	0.09	0.764	1.07	0.304
Sex	0.39	0.535	0.00	0.955
Baseline IOP	0.75	0.390	1.77	0.188
RVO type	0.11	0.744	0.10	0.754
RVO duration	0.91	0.344	1.15	0.288
Hypertension duration	0.00	>0.999	1.42	0.238
Model fit				
Conditional *R*^2^	0.987		0.791	
Marginal *R*^2^	0.603		0.779	

^†^FT, foveal thickness; BCVA, best corrected visual acuity; IOP, intraocular pressure, RVO, Retinal Vein Occlusion. Unit: FT, μm; BCVA, logMAR; age, years; IOP, mmHg; RVO duration, months; hypertension duration, years. ^§^Type-III *F* tests were computed using Satterthwaite denominator degrees of freedom from lmerTest on REML fits.

At the 1-month primary endpoint, the adjusted mean BCVA (logMAR) was significantly better in Group A (0.40, 95% CI: 0.31–0.48) and Group M (0.43, 95% CI: 0.35–0.51) compared to Group Z (0.74, 95% CI: 0.64–0.83). The pairwise differences were significant for Group A vs. Group Z (−0.34, 95% CI: −0.50 to −0.18, *p* < 0.001) and Group M vs. Group Z (−0.31, 95% CI: −0.46 to −0.16, *p* < 0.001), whereas Group A vs. Group M was null (−0.03, 95% CI: −0.17 to 0.11, *p* > 0.999). The adjusted mean foveal thickness (μm) was as follows: Group A, 299.0 (95% CI: 281.2–316.7); Group M, 311.3 (95% CI: 295.4–327.3); Group Z, 353.8 (95% CI: 333.9–373.7). The pairwise differences were significant for Group A vs. Group Z (−54.8, 95% CI: −87.4 to −22.2, *p* < 0.001) and Group M vs. Group Z (−42.5, 95% CI: −73.7 to −11.3, *p* < 0.001), but Group A vs. Group M was null (−12.3, 95% CI: −41.5 to 16.9, *p* = 0.93).

Our sensitivity analysis indicated an MDE of 0.18 LogMAR for BCVA and 38.1 μm for foveal thickness. This suggests that the study was robustly powered to detect the primary benefit of blood pressure management, while any true differences between full and partial BP control (Group A vs. Group M) likely fall below our margin of detectable clinical significance.

## Discussion

ME is the principal cause of vision impairment in RVO (20), and its successful management is the main goal of treatment (21). While traditional therapies like photocoagulation can be effective in controlling disease progression and neovascularization (22), anti-VEGF therapy has emerged as a new and highly effective means to directly improve visual acuity and become a cornerstone of modern treatment (11, 12, 23). However, clinical response to anti-VEGF agents is notably heterogeneous. Some patients respond well, while others suffer from persistent or recurrent edema despite repeated injections (24), indicating that factors beyond VEGF alone modulate the disease. Our results strongly suggest that systemic hypertension control is a key factor associated with RVO management. Patients with controlled (Group A) or partially controlled (Group M) hypertension achieved significantly better visual acuity and greater reductions in foveal thickness at 1 month compared to patients with persistently uncontrolled hypertension (Group Z). That is, systemic BP control is significantly associated with the therapeutic efficacy of intravitreal Conbercept for ME secondary to RVO.

RVO is mechanistically linked to systemic vascular diseases (13, 14), and hypertension is a well-established, independent risk factor for both the development of RVO and for a poorer long-term visual prognosis (2, 15, 25). We propose a “dual-assault” mechanism to interpret our findings. RVO triggers an acute ischemic cascade and VEGF upregulation (4, 5), leading to vascular permeability (6). Concurrently, chronic, uncontrolled hypertension contributes its own burden of microvascular damage by compromising endothelial function and integrity (7, 16). Hence, for patients in Group Z, the retinal vasculature is battling two distinct insults; for these patients, anti-VEGF therapy may be less effective because it only addresses the VEGF-driven component, leaving the underlying hypertensive vasculopathy untreated. Conversely, patients in Groups A and M, who had better BP control, likely have a more stable and functional endothelium. This healthier baseline state may reduce the overall level of non-VEGF-mediated leakage and make the retinal vasculature more responsive to Conbercept, leading to superior outcomes. This hypothesis is supported by case studies where systemic hypertension control alone was sufficient to resolve ME (17).

This study did not, however, find a statistically significant difference in outcomes between the fully controlled (Group A) and partially controlled (Group M) groups. Our sensitivity analysis confirms that this null finding must be interpreted with caution: the observed difference of 0.03 LogMAR in BCVA and 12.3 μm in foveal thickness between Groups A and M was below the minimal detectable effect threshold for our sample size (0.18 LogMAR and 38.1 μm, respectively). Therefore, we cannot exclude the possibility of a small but clinically meaningful benefit of strict versus partial BP control, which would require a larger study to detect. Furthermore, we did not stratify the types and dosages of antihypertensive drugs used by the patients, although the differences in clinical application of different antihypertensive drugs may affect the relationship between BP control and treatment efficacy. Nonetheless, the clinical implications are clear. Our findings advocate for the rigorous screening of systemic hypertension in all patients presenting with RVO. The management of RVO-related ME should not be an isolated ocular endeavor but rather a collaborative effort that includes systemic antihypertensive therapy, coordinated with an internal medicine specialist.

In summary, this study demonstrates that optimized systemic BP control is significantly associated with superior short-term visual and anatomical outcomes following Conbercept therapy for RVO-related ME. Determining if BP management effectively lowers the overall anti-VEGF treatment burden has significant implications for reducing patient costs, healthcare risks, and the overall burden of care. However, several limitations must be considered in interpreting these findings. First, the retrospective, single-center design and relatively small sample size may introduce selection bias. Because BP control was defined concurrently with the treatment period rather than pre-baseline, these findings represent a statistical association rather than a confirmed causal effect. Despite our use of multivariable adjustment, the possibility of residual confounding from lifestyle factors or unmeasured clinical variables remains. Second, the clinical management of hypertension was heterogeneous. Patients utilized various classes of antihypertensive medications prescribed by different providers, and our broad stratification did not capture the potential independent effects of specific drug classes (e.g., ACE inhibitors vs. calcium channel blockers) on vascular permeability. Third, we were unable to account for several critical ocular confounders. For example, due to the masking effect of extensive intraretinal hemorrhages on baseline FA, we could not reliably stratify patients into ischemic versus non-ischemic RVO subtypes. Fourth, our analysis focused on foveal thickness as the primary anatomical endpoint; we did not systematically evaluate nuanced OCT biomarkers such as ellipsoid zone integrity, hyperreflective foci, or the presence of subretinal fluid, which are known to influence long-term prognosis. Finally, the one-month follow-up period following a single injection is a significant constraint that does not align with the international standard of a three-month loading phase. Consequently, these results may not reflect long-term efficacy or the real-world treatment burden. Future prospective research with extended follow-up is required to investigate whether optimized systemic BP control can reduce the frequency of required intravitreal injections and improve the sustainability of visual gains.

## Data Availability

The original contributions presented in the study are included in the article/Supplementary material, further inquiries can be directed to the corresponding authors.
